# Outcomes of antiretroviral treatment for 0-14-year-old children living with HIV in Ganzhou, China, 2006–2023

**DOI:** 10.1186/s12981-024-00594-8

**Published:** 2024-01-31

**Authors:** Ting Zeng, Xin Chen, Xiao-Yi Zhang, Chao-Xian Lian, Rong-Rong Yang, Li-Ling Yu, Xiao-Kang Liao, Dan-Dan Huang, Yu-Ning Zhang, Hong-Min Cao

**Affiliations:** 1 Department of Pathogenic Biology, School of Basic Medical Sciences, Gannan Medical University, Ganzhou, China; 2 Department of Epidemiology, School of Public Health and Health Management, Gannan Medical University, Ganzhou, China; 3 Department of AIDS/STD Control and Prevention, Ganzhou Center for Disease Control and Prevention, No. 6 Zhangjiangbei Avenue, Zhanggong District, Ganzhou, 341000 China

**Keywords:** HIV, Antiretroviral therapy, Children, Outcome, Ganzhou

## Abstract

**Background:**

Studies on antiretroviral therapy (ART) in children living with HIV (CLHIV) are limited due to the small population and low accession rate of ART.

**Methods:**

All 0-14-year-old CLHIV admitted to the Ganzhou Center for Disease Control and Prevention from January 2006 to June 2023 were included retrospectively. The information of treatment regimens, disease progression, and laboratory tests of the patients under ART were used to explore the outcomes and impacts of long-term ART. The normality of all the data was tested by the Shapiro-Wilk test.

**Results:**

From 2006 to 2023, 18 CLHIV were reported in Ganzhou. Among them, 11 received ART and were followed up for 60.0 ± 48.4 months. After receiving ART, the median viral load of them decreased from 89,600 copies/ml to 22 copies/ml (*P* = 0.007), the median CD4^+^ T cell count increased from 380.7 cells/µL to 661.9 cells/µL (*P* = 0.028), and the median CD8^+^ T cell count decreased from 1065.8 cells/µL to 983.3 cells/µL (*P* = 0.584). The laboratory test results regarding liver function, renal function, blood cell count, and glucolipid metabolism tended to be within normal reference ranges, and the mean height-for-age z-score and weight-for-age z-score increased. However, all the three CLHIV who received cotrimoxazole developed pneumocystis carinii pneumonia, upper respiratory infection, skin lesions, bacterial pneumonia and/or thrush; the mean body-mass-index-for-age z-score decreased from 0.52 to -0.63.

**Conclusion:**

For CLHIV, ART could effectively inhibit the replication of HIV and improve the immune function of patients. More studies that focus on ART in CLHIV are urgently needed.

## Introduction

The first case of human immunodeficiency virus (HIV) in China was reported in 1985 [1]. According to the Chinese Center for Disease Control and Prevention (CDC), the newly reported HIV infections increased from 13,258 in 2004 to 104,838 in 2018. Among them, the number of 0-14-year-old children increased from 212 in 2004 to 1,592 in 2018 [2]. Previous studies showed that over three-quarters of children living with HIV (CLHIV) in China were infected through mother-to-child transmission (MTCT) [3]. CLHIV experienced faster disease progression and higher mortality than adults due to underdeveloped physiological functions [4]. Without antiretroviral therapy (ART), more than one-third of them would die in infancy, and more than half of them would die under the age of two years [5].

ART suppressed viral replication, extending the life expectancy of people living with HIV by promoting immune reconstitution [6]. Early initiation of ART significantly decelerated AIDS progression and reduced the risk of death among people living with HIV [7, 8]. After early ART, the mortality of CLHIV decreased by 76% within two years, and the progression of AIDS slowed by 75% [9]. However, the rate of 0-14-year-old CLHIV who received ART (57%) was much lower than that of adults living with HIV (aged 15 years and older) (77%) [10]. Moreover, the group of children with HIV was much smaller than that of adults, which limited the studies on ART in CLHIV.

Most 0-14-year-old CLHIV were infected during the perinatal period, with incomplete immune function and drug metabolism ability [11]. Compared with adults living with HIV, they had higher baseline viral loads and required prolonged ART [12]. However, the efficacy and toxicity of long-term ART in CLHIV were not clear. Thus, studies exploring the outcomes and impacts of long-term ART in CLHIV are urgently needed to provide theoretical guidance for better treatment.

## Methods

### Ethics approval and consent to participate

The protocol of this study was approved by the Ethics Committee of Gannan Medical University (approval number: 2,021,320; approval date: October 5, 2021). All participants gave written informed consent.

### Population and data collection

Locating in eastern China, Ganzhou is the largest prefecture of Jiangxi Province, with nearly nine million resident population. In this study, all 0-14-year-old CLHIV who were diagnosed at the Ganzhou CDC from January 2006 to June 2023 were retrospectively included. Information on socio-demographic, follow-up medication, and laboratory test results of all participants available in the China Information System for Disease Control and Prevention were exported and explored.

According to the *Chinese Guidelines for Diagnosis and Treatment of HIV/AIDS* before 2015, CLHIV who initiated ART should meet one of the criteria: (1) 0-1-year-old; (2) 1-3-year-old with CD4^+^ T cell count less than 750 cells/µL; (3) 3-14-year-old with CD4^+^ T cell count less than 350 cells/µL. After 2015, CLHIV could receive ART voluntarily without any limitation of CD4^+^ T cell count. For people living with HIV, the criteria for receiving cotrimoxazole was with CD4^+^ T cell count less than 200 cells/µL. In this study, the basic information and outcomes of ART of CLHIV who received ART were explored.

### Statistical analysis

After testing by the Shapiro-Wilk test, the quantitative data with a normal distribution were described using the means ± standard deviation. In contrast, the quantitative data with a nonnormal distribution were described using the medians (percentage 25, percentage 75). Qualitative data were described using numbers and percentages.

The growth of the study children was measured by height-for-age z-score (HAZ), weight-for-age z-score (WAZ), and body-mass-index-for-age z-score (BAZ) based on the World Health Organization (WHO) growth reference data [13]. Since the frequency of laboratory tests varied, the last recorded laboratory test result before ART was considered the pre-ART result, and the last recorded laboratory test result during ART (as of June 2023) was considered the post-ART result. Test values exceeding sensitivity were substituted with sensitivity limits. Differences between the two results were analyzed using a paired t-test or paired rank sum test, depending on the normality of the data distribution. All statistical analyses were performed with the software Statistical Package for Social Sciences (SPSS, version 26, IBM Corporation, Armonk, USA) with a 95% confidence interval. A two-sided *P* value less than 0.05 was considered statistically significant.

## Results

### Characteristics of the study population

From January 2006 to June 2023, a total of 18 0-14-year-old CLHIV who resident in Ganzhou were diagnosed at Ganzhou CDC. Among them, 17 were outpatients with MTCT and one was inpatient with heterosexual transmission; six (33.3%) were males and twelve (66.7%) were females; 11 received ART and seven did not. The mortality rate within two years of diagnosis among patients who received ART was significantly lower than that among patients who did not receive ART (9.1% versus 57.1%, *P* = 0.047).

All 11 0-14-year-old CLHIV receiving ART were enrolled in the study (Table 1). From the results of the first laboratory tests, the mean age of these patients was 73.2 ± 39.6 mouths, the mean HAZ was − 2.28 ± 2.33, the WAZ was − 1.09 ± 0.92, the mean BAZ was 0.39 ± 1.27, the median CD4^+^ T cell count was 120 (41, 605) cells/µL and the median viral load was 54,600 (2593, 331,250) copies/mL. Among them, 72.7% were WHO clinical stage I and II, and 45.5% had opportunistic infections before starting ART, such as upper respiratory infection, skin lesions, and pneumocystis carinii pneumonia.

**Table 1:** Characteristics of 0-14-year-old children living with HIV at the start of ART in Ganzhou, 2006–2023

Patient no.	Gender	HIV transmission mode	Age (years)	Height (cm)	Weight (kg)	CD4 level (cells/µL)	Viral load (copies/mL)	WHO clinical stage	AIDS-related opportunistic infections	Cotrimoxazole
GZ5F1	Female	MTCT	5	110	18.0	110	1,790,000	I	-	Not
GZ5F2	Female	MTCT	5	92	16.0	1292	19,600	I	-	Not
GZ8F3	Female	MTCT	8	130	25.0	605	1340	II	-	Not
GZ8F4	Female	MTCT	8	122	23.0	101	89,600	II	-	Not
GZ13F5	Female	HT	13	150	43.0	306	158,000	II	Skin lesions	Not
GZ1M6	Male	MTCT	1	65	8.0	120	4230	IV	Pneumocystis carinii pneumonia; Upper respiratory infection	Access
GZ2M7	Male	MTCT	2	66	8.0	13	851,000	II	Pneumocystis carinii pneumonia; Upper respiratory infection	Not
GZ4M8	Male	MTCT	4	97	15.0	41	/	III	Skin lesions; Upper respiratory infection	Access
GZ5M9	Male	MTCT	5	95	11.5	19	< 20	III	Bacterial pneumonia; Thrush	Access
GZ6M10	Male	MTCT	6	100	18.0	224	3010	I	-	Not
GZ8M11	Male	MTCT	8	130	24.0	1017	112,000	I	-	Not

HT, heterosexual transmission; MTCT, mother-to-child transmission; /, the patient did not test for viral load; -, the patients did not present with any AIDS-related opportunistic infections

### Treatment and follow-up situation

The median duration of diagnosis to first ART among the 11 0-14-year-old CLHIV was 14 months, ranging from 32 days to six years (Table 2). Ten of them (90.9%) received a nationally approved triple-drug regimen in China and one of them received only Stavudine (D4T). Six of them (54.5%) took lamivudine (3TC), zidovudine (AZT), and nevirapine (NVP) as their first treatment regimen.

**Table 2:** The treatment of 0-14-year-old children living with HIV in Ganzhou, China

Patient no.	Duration of diagnosis to first ART (months)	Initial ART regimen	Follow-up duration(months)	AIDS-related opportunistic infections	Cotrimoxazole	Current ART regimen	Current status
GZ5F1	32	3TC/AZT + NVP	77	-	Not	3TC/AZT + NVP	Follow-up
GZ5F2	27	3TC + AZT + EFV	89	-	Access	3TC + AZT + EFV	Follow-up
GZ8F3	1	3TC + ABC + EFV	31	-	Not	3TC/AZT + EFV	Follow-up
GZ8F4	71	3TC + AZT + NVP	34	-	Access	3TC/AZT + NVP	Follow-up
GZ13F5	3	3TC + TDF + EFV	28	Skin lesions	Access	3TC + TDF + EFV	Follow-up
GZ1M6	3	3TC + AZT + NVP	124	Bacterial pneumonia; Pneumocystis carinii pneumonia	Not	3TC + AZT + NVP	Follow-up
GZ2M7	4	3TC + AZT + NVP	116	-	Access	3TC + AZT + LPV/r	Follow-up
GZ4M8	14	D4T	1	-	Not	D4T	Dead
GZ5M9	2	3TC + AZT + NVP	129	Bacterial pneumonia	Not	3TC/AZT + NVP	Follow-up
GZ6M10	69	3TC + AZT + NVP	28	-	Not	3TC + AZT + NVP	Follow-up
GZ8M11	20	3TC + AZT + EFV	3	-	Not	3TC + AZT + EFV	Loss to follow-up

3TC, lamivudine; ABC, abacavir; AZT, zidovudine; D4T, stavudine; EFV, efavirenz; LPV/r, lopinavir/ritonavir; NVP, nevirapine; TDF, tenofovir; -, the patients did not present with any AIDS-related opportunistic infections. The current treatment regimens for deceased and lost-to-follow patients were the final treatment regimens before death or loss of follow-up

The 11 CLHIV were followed up for one month to 11 years after starting ART, with a mean duration of 60.0 ± 48.4 months (Table 2). During the follow-up period, the mean rate of lost to follow-up, stopped-taking medicine, and missed doses was 3.1%, 3.2%, and 4.2%, respectively. Four patients took cotrimoxazole, and one of them had skin lesion, while among the seven patients who did not take cotrimoxazole, two of them had bacterial pneumonia and pneumocystis carinii pneumonia. The opportunistic infection rates between these two groups were not significantly different (*P* = 0.721). However, compared with the period prior to ART, the opportunistic infection rate decreased rapidly after one month of ART. During the treatment, patient GZ8F3 changed her regimen due to drug interaction, and patient GZ3M7 switched to the second-line regimen due to virological failure.

As of 2023, one patient died, one patient was lost to follow-up. For the nine patients who had been followed up for more than 28 months, the mean HAZ increased from − 2.61 ± 2.40 to -0.58 ± 1.62, and the mean WAZ increased from − 1.20 ± 1.01 to -0.25 ± 1.15. However, compared to the beginning of ART, the mean BAZ decreased from 0.52 ± 1.34 to -0.63 ± 1.04 in the last follow-up of this study.

### Laboratory results

The changing trends of viral load, CD4^+^ T cell count, and CD8^+^ T cell count were explored among nine 0-14-year-old CLHIV who were followed up for more than 28 months in this study (Table 2). After receiving ART, the patient’s viral load decreased quickly and then stabilized at a low level, from 89,600 (4230, 851,000) copies/ml at the beginning of ART to 22 (20, 45,500) copies/ml at the last time of follow-up (*P =* 0.007). The CD4^+^ T cell count of the CLHIV increased in the early stages of ART and then remained stable, from 380.7 ± 447.4 cells/µL at the beginning of ART to 661.9 ± 316.2 cells/µL at the last time of follow-up (*P =* 0.028). The CD8^+^ T cell count showed insignificant changing trends, from 1065.8 ± 687.2 cells/µL at the beginning of ART to 983.3 ± 311.0 cells/µL at the last time of follow-up (*P =* 0.584).

After receiving ART, the white blood cell count and the plasma creatinine significantly increased (*P* < 0.05); the platelet count, hemoglobin, triglycerides, total cholesterol, glucose, alanine aminotransferase, and total bilirubin slightly increased (*P* > 0.05), while the aspartate aminotransferase slightly decreased (*P* > 0.05).

## Discussion

In this study, the ART acquisition rate among 0-14-year-old CLHIV in Ganzhou from 2006 to 2023 was 61.1%. The mortality rate in children without ART was significantly higher than in children with ART (*P* = 0.047). Most CLHIV achieved viral suppression within the first year of ART and regained partial immune function, with fewer opportunistic infections. In addition, we further used growth metrics (HAZ, WAZ, and BAZ) to evaluate the treatment effect of ART in 0-14-year-old CLHIV and found that growth recovery was not significant in them. These findings suggest that ART can reduce mortality and slow the progression of the disease in 0-14-year-old CLHIV. Therefore, CLHIV should be diagnosed and treated as early as possible. During treatment, doctors and treatment organizations should pay attention to the children’s growth indicators and formulate appropriate nutritional support programs to help CLHIV achieve the best possible therapeutic outcomes.

Among the WHO-recommended first-line antiretroviral regimens for CLHIV, the non-nucleoside drug NVP was favored in resource-limited areas due to its heat resistance, fixed-dose combinations, affordability, and safety [14]. Several studies had shown that NVP-based ART lowers clinical events in children, but older children and those with higher CD4 counts may develop a rash [15, 16]. In this study, 54.5% of the CLHIV chose NVP-based ART, with 66.7% achieving complete viral suppression in 3–16 months, but some had bacterial pneumonia. Extended NVP exposure led to high resistance in perinatally infected patients [17, 18]. In the present study, patient GZ2M7 with primary NVP resistance had unsuppressed viral load and low CD4^+^ T cell count while on the NVP regimen but switching to a Lopinavir/ritonavir-based regimen improved viral suppression and CD4^+^ T cell count. Consequently, for maternal women living with HIV and HIV perinatally infected children, drug resistance testing before initiating ART might provide useful information to develop optimal treatment regimens.

Previous studies had shown that the toxic effects of ART can lead to hepatocellular damage, renal abnormalities, and disorders of glucolipid metabolism in adult patients [15, 19]. However, the toxic effects in pediatric patients were unknown. This study innovatively used blood, renal function, glucolipid metabolism, and liver function parameters to evaluate drug toxicity in CLHIV undergoing ART for a prolonged period. The results showed that the ART’s impact on CLHIV’s blood cell counts and glucolipid metabolism were transient and resolved spontaneously, and the function of the renal and kidney gradually improved and stabilized as the ART duration was prolonged. Study has revealed that cotrimoxazole significantly improves the survival rate of adult people living with HIV by reducing the risk of contracting HIV-related diseases [20]. Our study found that cotrimoxazole before ART did not prevent infections effectively but reduced opportunistic infections during ART. These findings suggest potential differences in antiviral regimen toxicity between pediatric and adult patients, warranting further in-depth research.

## Conclusion

In the present study, the outcomes and impacts of long-term ART were explored by comparing the differences in the treatment regimen, disease progression, and laboratory test results among 11 0-14-year-old CLHIV in Ganzhou, China. The results showed that ART could effectively inhibit the replication of HIV and improve the immune function, liver function, renal function, blood cell count, and glucolipid metabolism of the patients. However, the administration of cotrimoxazole prior to ART initiation did not effectively prevent opportunistic infections, and the recovery of growth metrics in children is not significant. More studies focusing on ART in CLHIV are urgently needed to help them achieve better outcomes.

## Data Availability

The data supporting this study’s findings are available from the corresponding author (2977560371@qq.com) upon reasonable request.

## Abbreviations

3TC: Lamivudine
ART: Antiretroviral therapy
AZT: Zidovudine
BAZ: Body-mass-index-for-age z-score
CDC: Center for Disease Control and Prevention
D4T: Stavudine
EFV: Efavirenz
HAZ: Height-for-age z-score
HIV: Human immunodeficiency virus
LPV/r: Lopinavir/ritonavir
MTCT: Mother-to-child transmission
NVP: Nevirapine
WAZ: Weight-for-age z-score
WHO: World Health Organization
